# Anxiety, Distress and Stress among Patients with Diabetes during COVID-19 Pandemic: A Systematic Review and Meta-Analysis

**DOI:** 10.3390/jpm12091412

**Published:** 2022-08-30

**Authors:** Rubén A. García-Lara, José L. Gómez-Urquiza, María José Membrive-Jiménez, Almudena Velando-Soriano, Monserrat E. Granados-Bolivar, José L. Romero-Béjar, Nora Suleiman-Martos

**Affiliations:** 1 UGC Orgiva, Granada-South Health Management Area, Andalusian Health Service, Calle La Madre s/n, Lanjarón, 18420 Granada, Spain; 2 Faculty of Health Sciences, University of Granada, Cortadura del Valle s/n, 51001 Ceuta, Spain; 3 Red Cross Nursing Center, University of Sevilla, Av. la Cruz Roja, 41009 Sevilla, Spain; 4 Virgen de las Nieves University Hospital, Av. de las Fuerzas Armadas, 18014 Granada, Spain; 5 UGC Iznalloz, Granada Metropolitan District, Andalusian Health Service, Calle Virgen de la Consolación, 12, 18015 Granada, Spain; 6 Department of Statistics and Operations Research, University of Granada, Avda. Fuentenueva s/n, 18071 Granada, Spain; 7 Instituto de Investigación Biosanitaria (ibs.GRANADA), 18012 Granada, Spain; 8 Institute of Mathematics, University of Granada (IMAG), Ventanilla 11, 18001 Granada, Spain; 9 Faculty of Health Sciences, University of Granada, Avenida de la Ilustración, 60, 18016 Granada, Spain

**Keywords:** anxiety, COVID-19, diabetes, distress, meta-analysis, stress

## Abstract

The prevalence of mental health disorders has increased during the COVID-19 pandemic. Patients with chronic diseases, such as diabetes, are a particularly vulnerable risk group. This study aims to assess the levels and prevalence of anxiety, distress, and stress in patients with diabetes during the COVID-19 pandemic. A systematic review was conducted in CINAHL, Cochrane, LILACS, Medline, SciELO, and Scopus in accordance with the Preferred Reporting Items for Systematic Reviews and Meta-Analyses (PRISMA) statement. Thirty-seven articles with a total of 13,932 diabetic patients were included. Five meta-analyses were performed. The prevalence of anxiety was 23% (95% CI = 19–28) in T1DM and 20% (95% CI = 6–40) in T2DM patients. For diabetes distress it was 41% (95% CI = 24–60) for T1DM and 36% in T2DM patients (95% CI = 2–84). For stress, the prevalence was 79% (95% CI = 49–98) in T1DM patients. People with diabetes have significant psychiatric comorbidity as well as psychological factors that negatively affect disease management, increasing their vulnerability in an emergency situation. To establish comprehensive care in diabetic patients addressing mental health is essential, as well as including specific policy interventions to reduce the potential psychological harm of the COVID-19 pandemic.

## 1. Introduction

The coronavirus infection (COVID-19) has become a global health problem since the beginning of 2020 [1]. The lockdown as well as the restrictions in the different waves of contagion have caused a negative impact on the health of the general population and especially on people who suffer from chronic diseases such as people with diabetes [2]. People with diabetes mellitus (DM) are a risk group, with high hospitalization and mortality rate, and this risk increases when there is COVID-19 infection [3].

The prevalence of mental health disturbances has increased at an alarming rate during the COVID-19 pandemic [4]. Patients with DM present multiple psychosocial factors, which together with the psychological stressors of a pandemic, such as quarantine, social distance, and fear of contagion, make this group even more vulnerable [5]. Mental disorders in DM patients reach figures of up to 50%, which predisposes to an increase in mental health disorders in the face of a pandemic situation that leads to difficulties in adapting psychologically [6]. Some reports show that up to 87% of DM type 2 patients indicate being “psychologically affected” [7].

Among the possible issues in psychological health, we can find a greater susceptibility to severe symptoms of depression and a feeling of loneliness, anxiety, stress, or diabetes stress, referring to negative emotions related to the disease such as feeling frustrated, desperate, or angry [8,9,10]. These comorbidities in DM patients can reduce self-care, adherence to treatment and engagement with health professionals, with a negative impact on disease management [11,12]. Several studies indicate that up to 50% of DM patients were afraid of possible contagion [7]. This situation, together with medical distrust, and frustration due to the difficulties in DM management, is related to a reduction in control visits and even more in the demand for assistance in non-emergencies problems, especially those related to mental health [13,14].

The lockdown and successive waves of restrictions have disrupted healthy lifestyle patterns and the ability to self-care [14]. Some studies report that up to 54% of chronic patients claim to have problems related to their usual treatment [15], and data from a survey conducted in 155 countries by the World Health Organization showed that diabetes treatment was partially or completely interrupted in 49% of the countries surveyed [16]. Unhealthy behaviours in DM patients with higher consumption of sugary drinks as well as a reduction in physical activity have also been reported [7]. Other studies report a reduction in self-monitoring of blood glucose; only 28% of patients regularly monitored glucose levels during the COVID-19 lockdown [17]. Given these data, some authors show a clear relationship between self-care deficit and an increase in the number of mental disorders [18].

Although there are several studies that analyse mental health in the general population, data about chronic disease patients and more specifically in patients with DM are still limited. There are studies focused on the treatment of diabetes and associated complications during the COVID-19 pandemic [5,19,20]; however, no systematic review and meta-analysis address psychological disturbances.

An analysis of levels of these variables, looking at the definition by the Medical Subject Headings, anxiety (“feelings or emotions of dread, apprehension and impending disaster”), distress (“negative emotional state with emotional and/or physical discomfort”), and stress (with emotional factors predominating) in the population with DM is necessary, since the number of DM patients affected by these problems before the COVID-19 pandemic was important [21] and these levels may have increased. This review analyses the data currently available in the pandemic scenario, in order to establish intervention strategies and address a psychosocial approach in people with DM during COVID-19. Therefore, the objective of this systematic review and meta-analysis was to analyse the levels and prevalence of anxiety, distress, and stress during the COVID-19 pandemic in diabetic patients.

## 2. Methods

### 2.1. Design

The review and meta-analysis were reported according to the preferred reporting items for systematic reviews and meta-analyses (PRISMA) guidelines [22] (see Supplementary Materials Table S1 for further information). The protocol was registered in PROSPERO (International Prospective Register of Systematic Reviews) with the registration number CRD42022325197.

### 2.2. Search Strategy

A search was performed in the following databases: the Cumulative Index to Nursing and Allied Health Literature (CINAHL) (EBSCO), the Cochrane Central Register of Controlled Trials (CENTRAL), LILACS (BIREME), Medline (Ovid), SciELO (BIREME), and Scopus (Elsevier). The search was done in July 2022 without restriction by language or publication date. The search terms used were: “(anxiety OR psychological distress OR stress) AND (diabetes OR chronic illness OR chronically ill OR non-communicable diseases) AND (SARS-CoV-2 OR coronavirus OR COVID-19)”.

### 2.3. Eligibility Criteria

Studies conducted during the COVID-19 pandemic were included with the following inclusion criteria: (1) original studies, (2) type 1 diabetes mellitus (T1DM) or type 2 diabetes mellitus (T2DM), (3) assessing anxiety, distress, or stress symptoms (percentages, means, or median levels), (4) use of anxiety, distress, and stress validated measurement tool. There was no restriction by language or publication date.

Studies were excluded if they were: (1) letters to editors, conference paper review articles, and case reports, (2) articles with other types of diabetes (gestational, MODY, LADA), (3) articles including different chronic pathologies without indicating a number of participants with diabetes, (4) sample of patients with serious cognitive/neurological impairment or mental/physical disability.

### 2.4. Study Selection and Data Collection

**Figure 1:**
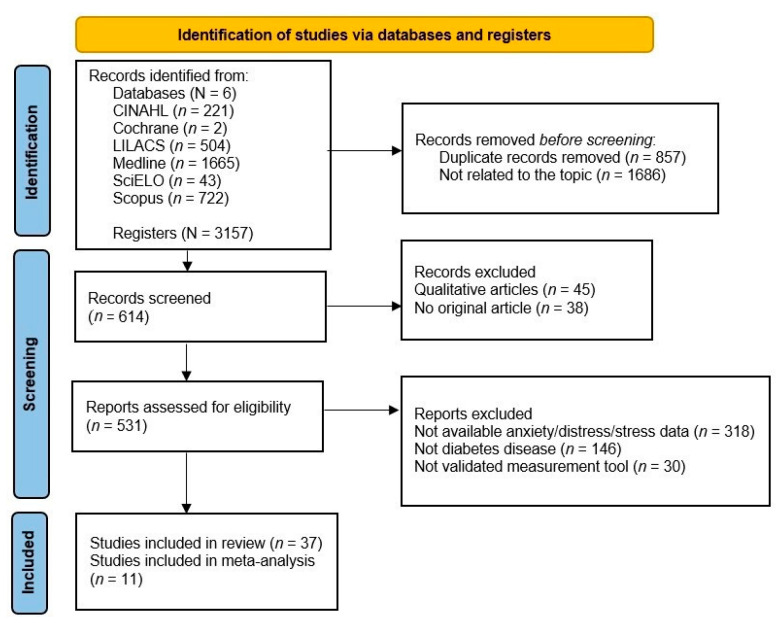
Flow diagram of the selection process.

First, two independent reviewers analysed titles and abstracts and then the full texts according to the inclusion criteria (Figure 1). A third author was consulted in case of disagreement.

Two authors extracted data from selected studies into an Excel spreadsheet, consulting with a third author in case of discrepancies. The following information was extracted from each study: (1) author, year of publication, country, (2) study design and period, (3) sample, (4) setting, (5) measuring instrument, (6) type of diabetes, (7) levels of anxiety, distress, or stress (percentage, mean, median) (Table 1).

### 2.5. Quality Assessment, Evidence Level and Grade of Recommendation

A quality assessment and bias analysis were carried out by two reviewers independently with a third reviewer consulted in case of disagreement.

The National Heart, Lung and Blood Institute quality assessment scale was used for bias assessment of observational studies [23] (Appendix A). The recommendations of the OCEBM were also used (Oxford Centre for Evidence-Based Medicine) to analyse the levels of evidence and grades of recommendation [24] (Table 1).

### 2.6. Data Analyses

A descriptive analysis was performed for the systematic review, extracting the variables in a data table.

For the meta-analysis, all the studies that presented data on the percentage of anxiety, diabetes distress, or stress measured through the same tool were used. Heterogeneity was assessed using the I^2^ index. Random effects meta-analysis were performed [25]. Sensitivity analysis and Egger’s regression test were used to assess bias in the studies.

Five meta-analyses were performed to estimate the prevalence of anxiety, diabetes distress or stress, and the corresponding confidence interval. StatsDirect software (StatsDirect Ltd., Cambridge, UK) was used for all statistical calculations.

## 3. Results

### 3.1. Characteristics of the Studies Included

The initial search found 3157 results. After deleting duplicates and reading the title and abstract, a total of 614 articles were selected. Finally, after reading the full text and analysing the inclusion criteria, 37 articles were included. The study search and selection process are shown in Figure 1.

All the studies found were observational (cross-sectional, retrospective, or prospective) and one was a case-control study. The total sample population consisted of 13,932 type 1 and type 2 diabetic patients. Most studies were conducted in Italy (*n* = 5), US (*n* = 5), followed by Saudi Arabia (*n* = 3), and Turkey (*n* = 3) (Table 1).

To measure anxiety, the most used questionnaires were the General Anxiety Disorder-7 (GAD-7) (*n* =7) and the Visual Analog Scale (VAS) for anxiety (*n* = 3). The remaining questionnaires used for anxiety were the Hospital Anxiety and Depression Scale (HADS), the Test of Depression and Anxiety Scale (TAD), Spence Children Anxiety Scale (SCAS), the Symptom Check List-revised anxiety subscale (SCL-ANX4), the General Health Questionnaire-12 items (GHQ-12), and the State-Trait Anxiety Inventory (STAI) (see Table 1).

The scales used to measure distress were the Diabetes Distress Scale (DDS) (*n* = 6), the Kessler Psychological Distress Scale (K10) (*n* = 3), the questionnaire Problem Areas in Diabetes-Distress item (PAID) (*n* = 3), and the Beirut Distress Scale (BDS22) (Table 1).

Finally, the stress measurement tools used were the Perceived Stress Scale (PSS) (*n* = 11), the Visual Analog Scale (VAS) for stress (*n* = 3), and the Impact of Event Scale Revised (IES-R) (Table 1). 

The data were collected in different settings that included the collection of information through telephone surveys, online forms or through face-to-face at outpatient clinics, hospitals, or primary care centres. Most of the studies (*n* = 21) collected data during the first phase of the pandemic (January–June 2020).

The studies included had an adequate level of quality; according to the measurement tools applied there were no exclusions. The assessment and characteristics of the studies are represented in Table 1.

**Table 1:** Characteristics of the included studies (*n* = 37).

Author, Year, Country	Study /Period	Sample	Setting	Scale	Type of Diabetes	Anxiety/Distress/Stress M(SD)/M (IQR)	EL/RG
Abdelghani et al., [26], 2021, Egypt	Cross-sectional June–September 2020	N = 200 Mean age 48.4 (13.7) Female 63 % Mean duration of DM 6.2 (5.3) years	Endocrinology outpatient clinic	HADS-Anxiety	T1DM T2DM	Anxiety 8.8 (4.4)	2b/B
Abdoli et al. [27], 2021, US, Brazil, and Iran	Cross-sectional April–June 2020	N = 1788 US (*n* = 1099) Brazil (*n* = 477) Iran (*n* = 212) Age >18 years Female 78.28%	Online survey	DDS	T1DM	Distress No/little/moderate US 86.6% Brazil 69.2% Iran 42.9% High US 13.40% Brazil 30.8% Iran 57.1%	2b/B
Agarwal et al. [28], 2020, India	Cross-sectional April–May 2020	N = 89 Mean age 19.61 (3.8) Female 48.3% Mean duration of DM 8.4 (5) years	Online survey	PSS	T1DM	Stress Low 42.7% Moderate 51.7% Severe 5.6%	2b/B
Ajele et al., [29], 2022, Nigeria	Cross-sectional April–July 2021	N = 223 Mean age 53.26 (11.05) Female 26%	Outpatient clinic	PAID-DDS	T1DM T2DM	Distress 60.61 (29.51)	2b/B
Alkhormi et al., [30], 2022, Saudi Arabia	Cross-sectional August–-February 2022	N = 375 Female 51.7%	Diabetic center + primary healthcare centers	GAD-7	T2DM	Anxiety Normal 52.8% Moderate-Severe 47.2%	2b/B
Alshareef et al. [31], 2020, Saudi Arabia	Cross-sectional May 2020	N = 394 Female 42.9%	Phone survey	K10	T2DM	Distress 9.78 (4.14)	2b/B
Alzubaidi et al. [32], 2022, United Arab Emirates	Cross-sectional February–July 2021	N = 206 Female 42.2% Mean age 58.7 (11.2) Mean duration of DM 15.7 (8) years	Phone survey	DDS	T2DM	Distress Low 85.9% Moderate 10.7% High 3.4%	2b/B
Bao [33], 2021, China	Cross-sectional January 2019–December 2020	N = 256 Range age 25-78 years Female 57.4%	Department of Endocrinology	PAID-DDS	T2DM	Distress 32.16 (12.13) Moderate 37.89% Severe 20.31%	2b/B
Barchetta et al. [34], 2020, Italy	Observational retrospective study March–April 2020	N = 50 Mean age of 40.7 (13.5) Female 38%	Diabetes outpatient clinics	PSS	T1DM	Stress Low 26% Moderate 60% Severe 14%	2b/B
Büyükbayram et al. [35], 2022, Turkey	Cross-sectional January–July 2021	N = 184 Mean age of 51.77 (15.07) Female 52.2%	Internal medicine clinic	PSS	T2DM	Stress 23.82 (8.34)	2b/B
Caruso et al. [36], 2021, Italy	Cross-sectional study February–March 2020	N = 48 Mean age 42.4 (15.9) Female 47.9%	Endocrinology unit	GHQ-12	T1DM	Anxiety 4.5 Mild 50%	2b/B
Chao et al. [37], 2021, US	Observational prospective study July–December 2020	N = 2829 Mean age 75.6 (6) Female 63.2%	Health center	GAD-7	T2DM	Anxiety 2.4 (3.5) Moderate/Severe 5%	2b/B
Cusinato et al. [38], 2021, Italy	Observational retrospective study March–April 2020	N = 117 Mean age 15.9 (2.3) Female 44% Mean duration of DM 7.9 (4.6) years	Pediatric Diabetes Unit	TAD-Anxiety	T1DM	Anxiety 7%	2b/B
Cyranka et al., [39], 2021, Poland	Cross-sectional March–May 2020	N = 49 Mean age 29.8 (8.9) Female 75.5% Mean duration of DM 16.2 (7.3) years	Outpatient clinic	STAI PSS	T1DM	Anxiety STAI 39.7 (11) Stress PSS 21 (4.1)	2b/B
Di Dalmazi et al. [40], Italy	Observational retrospective study February–March 2020	N = 76 Mean age 45 years Female 48.7% Mean duration of DM 22 years	Endocrinology and diabetes unit	PSS	T1DM	Stress 14.5 (9.8–20)	2b/B
Di Riso et al. [41], 2021, Italy	Cross-sectional May–June 2020	N = 71 Mean age 11 (2.26) year Female 46.6%	Pediatric Diabetes Unit	SCAS-Anxiety	T1DM	Anxiety 16.7%	2b/B
Elhenawy & Eltonbary, [42], 2021, Egypt	Cross-sectional March 2020	N = 115 Female 53.9%	Online survey	PSS	T1DM	Stress Low 0% Moderate 66.6% Severe 33.4%	2b/B
Hosomi et al. [43], 2022, Japan	Observational retrospective study April–May 2020	N = 34 Mean age 59.1 (16) Female 67.6% Diabetes duration 14.5 (16)	Department of Endocrinology	VAS-Stress	T1DM	Stress 6.7 (2.1)	2b/B
Huang et al. [44], 2022, China	Cross-sectional study July–September 2020	N = 286	Clinics	VAS- Anxiety	T2DM	Anxiety 5.3 (2.8)	2b/B
Kim et al. [45], 2022, US	Cross-sectional June–December 2020	N = 84 Mean age 68.46 (5.41) Female 54.76% Mean duration of DM 13.89 (7.53) years	Online survey	DDS	T2DM	Distress 1.35 (1.55) 0.63%	2b/B
Khari et al. [46], 2021, Iran	Cross-sectional September–December 2020	N = 427 Female 66%	Online survey	PSS	T1DM T2DM	Stress 31.69 (5.88)	2b/B
Madsen et al., [47], 2021, Denmark	Observational prospective study March 2020	N = 1366 Mean age 61.7 (12.8) Female 44.5%	Online survey	DDS SCL-ANX4	T1DM T2DM	Distress DDS 1.8 (1.00) Low 75.4% Moderate-High 24.6% Anxiety SCL-ANX4 0.5 (0.66) <10% risk of anxiety 80.5% 20% risk of anxiety 14.6% 30% risk of anxiety 3.6% 40% risk of anxiety 1.1% 45% risk of anxiety 0.2%	2b/B
Magliah et al. [48], 2021, Saudi Arabia	Cross-sectional June 2020	N = 65 Mean age 30 (7.88) Female 70.8% Mean duration of DM 17.67 (6.89) years	Online survey	GAD-7	T1DM	Anxiety None/minimal 56.9% Mild 24.6% Moderate 10.8% Severe 7.7%	2b/B
Munekawa et al. [49], 2021, Japan	Cross-sectional April–May 2020	N = 203 Mean age 67.4 (11.3) Female 37.9% Mean duration of DM 14.4 (10.1) year	Department of Endocrinology a	VAS-Stress	T2DM	Stress 6.0 (1.7)	2b/B
Miller et al. [50], 2022, US	Observational prospective study March 2020	N = 41 Range age 10.3–19.1 years	Online survey	GAD-7 PSS	T1DM	Anxiety GAD-7 4.43 (4.63) Stress PSS 2.51 (0.71)	2b/B
Musche et al., [51], 2021, Germany	Cross-sectional April–June 2020	N = 240 Age > 18 years Female 74.3%	Online survey	GAD-7	T1DM T2DM	Anxiety T1DM (*n* = 169) None/minimal 46.2% Mild 30.8% Moderate 17.2% Severe 5.9% T2DM (*n* = 74) None/minimal 45.9% Mild 27% Moderate 14.9% Severe 9%	2b/B
Myers et al., [52], 2021, US	Observational prospective study May–June 2020	N = 404 Mean age 51.46 years Mean duration of DM 40.21 (17.70) years	Online survey	GAD-7 DDS PSS	T1DM T2DM	Anxiety GAD-7 T1DM (*n* = 100) 6.81 (4.96) Low-Mild 74% Moderate-Severe 26% T2DM (*n* = 304) 5.68 (5.50) Low-Mild 75.99% Moderate-Severe 24.01% Distress DDS T1DM (*n* = 95) 2.61 (0.85) Low 30.53% Moderate 35.79% High 33.68% T2DM (*n* = 293) 2.43 (0.95) Low 37.88% Moderate 32.08% High 30.03% Stress PSS T1DM (*n* = 100) 17.59 (6.99) Low 32% Moderate 59% High 9% T2DM (*n* = 304) 15.82 (8.33) Low 43.09% Moderate 46.05% High 10.86%	2b/B
Olickal et al. [53], 2020, India	Cross-sectional July–August 2020	N = 350 Female 22%	Phone survey	K10	T2DM	Distress Low 67.4% Moderate 30% High 2.6%	2b/B
Naous et al. [54], 2022, Lebanon	Cross-sectional January–June 2021	N = 461 Median age 59 years Female 47.4% Median duration of DM 10 years	Hospitals and private clinics	K10	T2DM	Distress 26 (18-35) Well 27.4% Mild 19.1% Moderate 15.1% Severe 38.4%	2b/B
Nassar & Salameh, [55], 2021, Lebanon	Case-control study April–May 2020	N = 72 Mean age 65.5 (10.5) Female 48.6%	Phone survey	BDS22-Anxiety	T2DM	Anxiety 0.5 (1.1)	2b/B
Regeer et al. [56], 2021, Netherlands	Cross-sectional May 2020	N = 536 Mean age 65.9 (7.9) Female 46% Mean duration of DM 13.3 (8) years	Online survey	PSS VAS-Anxiety	T2DM	Stress PSS 12.98 (6.61) Anxiety VAS 4.2 (2.5)	2b/B
Ruissen et al. [57], 2021, Netherlands	Observational prospective study March–June 2020	N = 435 Female 42%	Online survey	PSS	T1DM T2DM	Stress 13.25 (6.45) Elevated 34.1%	2b/B
Sacre et al. [58], 2021, Australia	Observational prospective study April–May 2020	N = 450 Mean age 66 (9) Female 31% Mean duration of DM 12 years	Phone/Online survey	GAD-7 PAID-DDS	T2DM	Anxiety GAD-7 2 (1.7–2.3) Mild 16.4% Moderate-Severe 8.4% Distress PAID 9 (8–10) Severe 7.8%	2b/B
Shin et al. [59], 2021, Korea	Cross-sectional April–July 2020	N = 246 Mean age 73.8 (5.7) Female 59.3% Mean duration of DM 17.7 (8.8) years	Outpatient clinic	IES-R-Stress	T2DM	Stress 6.4 (6.6) Minimal 97.2% Mild 1.2% Moderate 1.2% Severe 0.4%	2b/B
Silveira et al. [60], 2021, Brazil	Cross-sectional May–July 2020	N = 436 North, Northeast, Central-West (*n* =118) Southeast (*n* = 273) South (*n* = 45) Mean age 30.52 (9.22) Female 83% Mean duration of DM 15.29 (9.79) years	Online survey	DDS	T1DM	Distress Brazilian regions North, Northeast, Central-West 2.72 (0.99) No/Little 64.6% Moderate/High 35.4% Southeast 2.38 (1) No/Little 70.8% Moderate/High 29.2% South 2.76 (1.13) No/Little 68.8% Moderate/High 31.2%	2b/B
Sisman et al. [61], 2021, Turkey	Cross-sectional	N = 304 Mean age 42.1 (15.5) Female 56% Mean duration of DM 10.3 (8.5) years	Online survey	HADS-Anxiety	T1DM T2DM	Anxiety T1DM 7.1 (3.6) 44.7% T2DM 7.5 (4.3) 46.6%	2b/B
Utli & Vural Doğru [62], 2021, Turkey	Cross-sectional December 2020–April 2021	N = 378 Mean age 52.37 (11.37) Female 37.3%	Endocrinology clinic + outpatients’ department	VAS-Anxiety VAS-Stress	T2DM	Anxiety VAS-Anxiety 7.32 (1.56) Stress VAS-Stress 7.06 (1.62)	2b/B

2b = evidence level from the OCEBM, B = recommendation grade from the OCEBM, BDS22 = Beirut Distress Scale, DDS = Diabetes Distress Scale, DM = Diabetes Mellitus, EL = Evidence level, GAD-7 = General Anxiety Disorder-7, GHQ-12 = General Health Questionnaire-12 items, HADS = Hospital Anxiety and Depression Scale, IES-R = Impact of Event Scale Revised, IQR = Interquartile range, K10 = Kessler Psychological Distress Scale, PAID = Problem Areas in Diabetes-Distress item, PSS = Perceived Stress Scale, RG = Recommendation grade, T1DM = Type 1 diabetes, T2DM = Type 2 diabetes, TAD = Test of Depression and Anxiety Scale, SCAS = Spence Children Anxiety Scale, SCL-ANX4 = Symptom Check List-revised anxiety subscale, SD = Standard deviation, STAI = State-Trait Anxiety Inventory, VAS = Visual Analog Scale.

### 3.2. Mean Levels of Anxiety, Distress and Stress

The average anxiety levels varied from minimal [37,44,47,50,56,58,61], to mild [26,36,52], to moderate [62], to severe [39]. For diabetes distress, the mean levels were low [31,45,47,55,58], moderate [33,52,60], and high [29,54]. The mean stress levels found ranged from minimal [50,56,57,59], moderate [35,39,40,43,49,52,62], and high [46].

### 3.3. Meta-Analysis

Five random effects meta-analyses were performed with a total of 1024 T1DM patients and 4238 T2DM patients.

For anxiety according to the GAD-7 tool, the prevalence found in T1DM patients for moderate and severe levels (GAD-7 ≥ 10 score) was 23% (95% CI = 19–28) with low heterogeneity (I^2^ = 0%). For T2DM patients, it was 20% (95% CI = 6–40) with high heterogeneity (I^2^ = 99%).

For diabetes distress measured with the DDS questionnaire, the prevalence found in T1DM patients for moderate and high levels (DDS > 2) was 41% (95% CI = 24–60) with high heterogeneity (I^2^ = 93%), and for T2DM patients 36% (95% CI = 2–84) with high heterogeneity (I^2^ = 99%).

Finally, stress levels measured with the PSS questionnaire showed a prevalence in T1DM patients for moderate and high levels (PSS ≥ 14) of 79% (95% CI = 49–98) with high heterogeneity (I^2^ = 97%). Egger’s test showed no publication bias, and no study was removed after sensitivity analysis.

**Figure 2:**
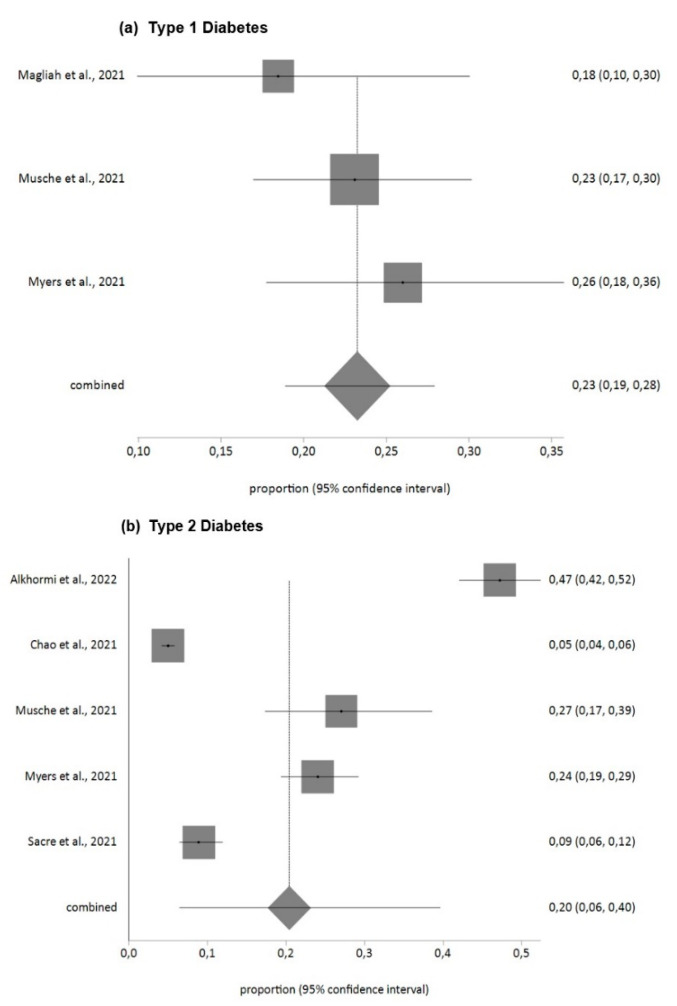
Prevalence of anxiety in DM patients during COVID-19 pandemic (GAD-7). (**a**) Type 1 Diabetes [48,51,52], (**b**) Type 2 Diabetes [30,37,51,52,58].

**Figure 3:**
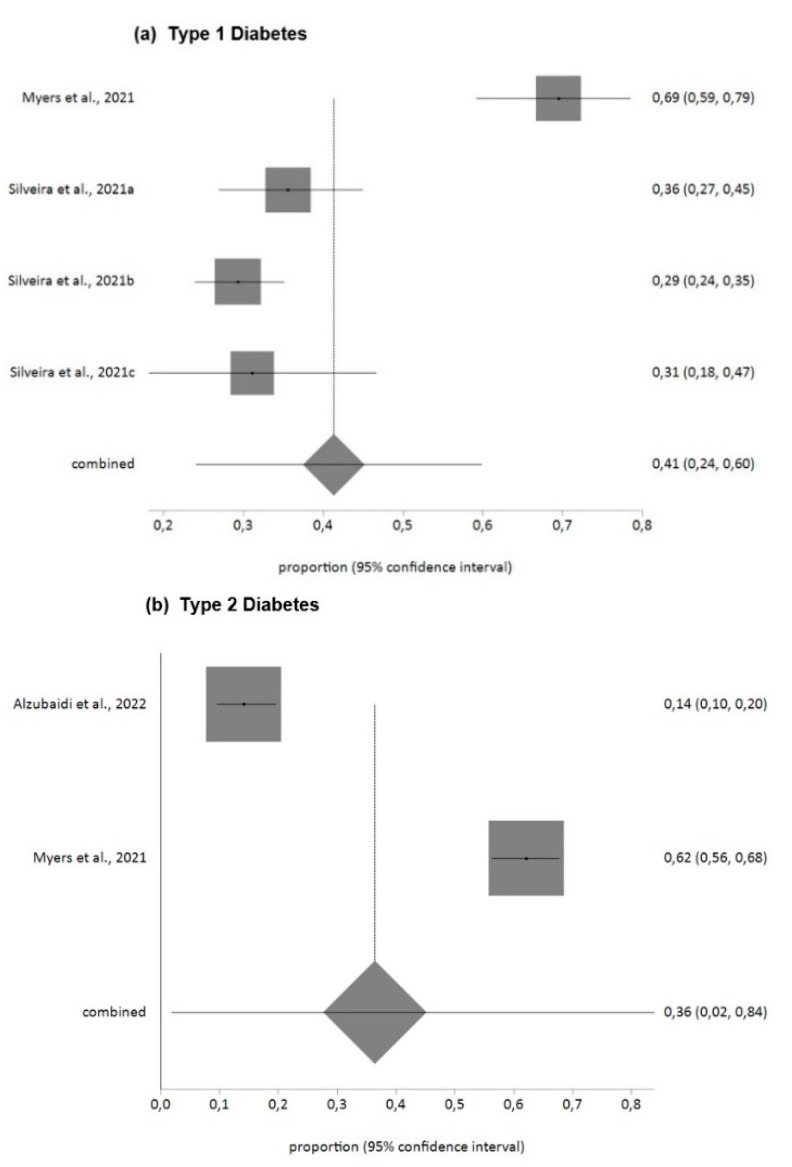
Prevalence of distress in DM patients during COVID-19 pandemic (DDS). (**a**) Type 1 Diabetes [52,60], (**b**) Type 2 Diabetes [32,52].

**Figure 4:**
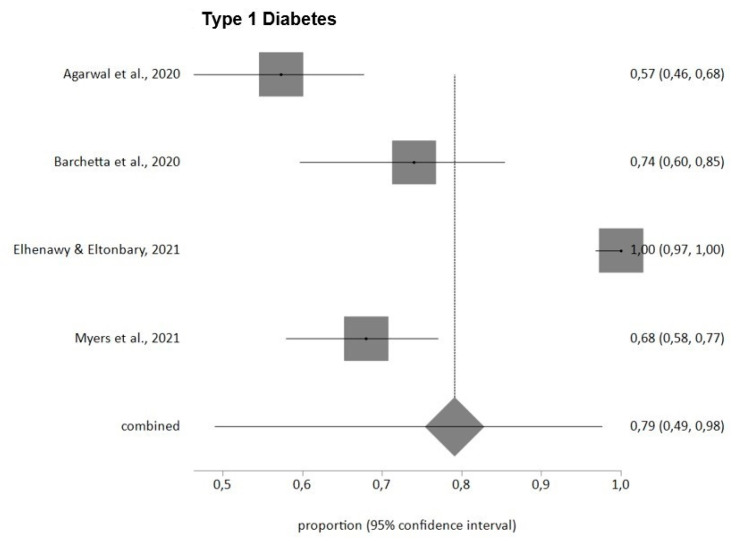
Prevalence of stress in DM patients during COVID-19 pandemic (PSS) [28,34,42,52].

Figure 2, Figure 3 and Figure 4 summarize the findings in relation to anxiety, distress and stress prevalence.

## 4. Discussion

This study suggests relevant data about psychological disorders in the diabetic population during the pandemic, with a meta-analytical prevalence estimation of anxiety of 23% in T1DM patients and 20% in T2DM patients, diabetes distress of 41% in T1DM and 36% in T2DM, and stress of 79% in T1DM.

Studies before the pandemic reported a prevalence of anxiety symptoms of 17.7% for T1DM patients [63], and of 18% for T2DM [64] being for diabetes distress of 42.1% in T1DM [65] and 29.4% in T2DM [66], and for the stress of 50% in T1DM [67]. These data suggest a significant increase in symptoms.

In addition, the prevalence of anxiety found in DM patients was higher than that of studies performed in other groups during the pandemic. In the elderly population, the prevalence of anxiety symptoms found ranged from 10.10% [68] to 21.6% of moderate/severe anxiety in general population [69,70]. Other studies in the general population stated DM as one of the main psychosocial problems with a prevalence of up to 40% [71]. Even a recent meta-analysis in the general population showed that the mean prevalence of anxiety and psychological stress was 38.1% and 37.5%, respectively [72].

More than half of the population with chronic pathology wished to have received additional information about the risks associated with their medical condition during the pandemic [15]. Several authors indicate that the provision of diabetes care was significantly disrupted during the pandemic [73], as corroborated by studies conducted in chronic patients where 52% of adults and 38% of children worsened their health condition during confinement [74].

During the pandemic, the psychological disorders of diabetic patients are often not recognized or underestimated, which can impair the quality of life and self-management of the disease [75]. Greater support for self-care is related to higher adherence to the expected regimen and life changes [12]; however, psychological stressors can have an adverse effect, for example in the loss of good glycaemic control [76].

This study suggests a higher prevalence of anxiety and stress diabetes in T1DM patients, as corroborated by other studies that found several factors related to worse mental health such as T1DM or the female gender [70,77]. Other factors such as age remain controversial; some studies reported worse data in younger patients [70,77,78,79,80], while for others the levels were higher in older age groups [81].

Regarding the negative results of the pandemic involving mental health, other related factors were the fear of contagion by COVID-19 [82,83] and COVID-19 anxiety syndrome [84]. Studies reported that up to 27.3% of people with DM experienced stress due to the spread of the COVID-19 pandemic and 20% experienced stress due to fear of drug shortages [85]. Even in hospitalized patients, stress levels reached up to 39.3% [75], being lower than those found in our meta-analysis.

Several studies highlight these facts as a reason for greater concern and related them to a reduced capacity in the provision of psychological support to this group [73]. Therefore, finding strategies to identify and reduce anxiety, distress, and stress, as well as multiple other possible disorders such as depression or loneliness should be a priority for diabetes services [86]. In this sense, several studies support the routine implementation of telemedicine [87], as well as increasing the capacity of primary care to provide telehealth services for diseases related to COVID-19 and for several other chronic medical conditions [88]. Studies that have used the telemedicine care model have found positive benefits, for example in a higher mean reduction in the HbA1c level compared with traditional care model [89], so it could also have positive results in the treatment of mental health disorders.

Although a large number of protocols have been developed to identify and recover people with DM infected by COVID-19, there is still a large gap in mental health care. Managing DM in the midst of the COVID-19 pandemic has proven to be a real challenge. To date little is known about how pandemics globally affect the psychosocial health of people with DM. This study is the first meta-analysis to provide an assessment of current levels of anxiety, distress, and stress since the onset of COVID-19 exclusively in patients with DM. It is necessary to clarify the current situation of mental health disorders in these patients in order to establish intervention strategies.

### 4.1. Limitations

This study has several limitations. First, the inclusion of T21DM and T2DM patients from different countries could increase the heterogeneity given the differences in the conditions of the health system, the management and follow-up of the disease, and also clinical variability in the percentage of female, type of diabetes, or measurement instrument. The heterogeneity in the meta-analyses were also high. However, the results of this study may allow understanding the impact of the pandemic on these patients as a start for future research. Another limitation is the inclusion of all the data since the start of the pandemic (different restrictions and waves of contagion), which could increase the heterogeneity. Finally, the different methods of data collection (by telephone, online, or face-to-face interviews) could lead to bias. This review has shown that there are important levels of anxiety, distress, and stress in people with diabetes during the COVID-19 pandemic. Future research should analyse which factors are related with these problems and how those levels can be reduced.

### 4.2. Implication for Practice and Research

The COVID-19 infection and confinement have a diverse impact on access to health services, psychosocial well-being, and self-management of people with diabetes, which must be contextualized to the responses and preparation of each country. Diabetes significantly increases the risk of emotional and behavioural disorders, especially in times of social crisis such as the one experienced with the COVID-19 pandemic [90]. Improving effective self-care behaviours that include healthy coping (healthy eating, being active, blood glucose control) are essential components to establishing optimal behaviour goals, which in turn will improve mental health outcomes [5]. Future research is needed to analyse the monitoring of levels as the pandemic progresses, as well as large multicentre longitudinal studies to avoid the above-mentioned limitations.

## 5. Conclusions

The prevalence found during the COVID-19 pandemic for anxiety ranged between 23% and 20%, for diabetes distress between 41% and 36%, and for stress it was 79%. People with diabetes have significant psychiatric comorbidity as well as psychological factors that negatively affect disease management, increasing their vulnerability in an emergency situation. To establish comprehensive care in diabetic patients addressing mental health is essential, as well as including specific policy interventions to reduce the potential psychological harm of the COVID-19 pandemic. Moreover, assessing the variables that can prevent or reduce the development of anxiety, distress, and stress in this population would be important.

## Data Availability

Not applicable.

## Supplementary Materials

The following supporting information can be downloaded at: https://www.mdpi.com/article/10.3390/jpm12091412/s1, Supplementary Material Table S1: PRISMA 2020 checklist.

## Appendix A

**Table A1:** Observational studies quality assessment with National Heart, Lung, and Blood Institute.

Study	Q1	Q2	Q3	Q4	Q5	Q6	Q7	Q8	Q9	Q10	Q11	Q12	Q13	Q14
Abdelghani et al., [26], 2021, Egypt	Y	Y	Y	Y	Y	NA	NA	NA	Y	NA	Y	NA	Y	Y
Abdoli et al. [27], 2021, US, Brazil, and Iran	Y	Y	Y	Y	N	NA	NA	NA	Y	NA	Y	NA	NA	Y
Agarwal et al. [28], 2020, India	Y	Y	NR	Y	N	NA	NA	NA	N	NA	Y	NA	NA	Y
Ajele et al., [29], 2022, Nigeria	Y	Y	Y	Y	N	NA	NA	NA	Y	NA	Y	NA	NA	N
Alkhormi et al., [30], 2022, Saudi Arabia	Y	Y	Y	Y	Y	NA	NA	NA	Y	NA	Y	NA	NA	Y
Alshareef et al. [31], 2020, Saudi Arabia	Y	Y	NR	Y	N	NA	NA	NA	Y	NA	Y	NA	NA	Y
Alzubaidi et al. [32], 2022, United Arab Emirates	Y	Y	Y	Y	N	NA	NA	NA	Y	NA	Y	NA	NA	N
Bao [33], 2021, China	Y	Y	Y	Y	N	NA	NA	NA	Y	NA	Y	NA	NA	Y
Barchetta et al. [34], 2020, Italy	Y	Y	NR	Y	N	NA	NA	NA	Y	NA	Y	NA	NA	Y
Büyükbayram et al. [35], 2022, Turkey	Y	Y	NR	Y	Y	NA	NA	NA	Y	NA	Y	NA	NA	Y
Caruso et al. [36], 2021, Italy	Y	Y	Y	Y	N	NA	NA	NA	Y	NA	Y	NA	NA	N
Chao et al. [37], 2021, US	Y	Y	Y	Y	N	NA	NA	NA	Y	NA	Y	NA	NA	Y
Cusinato et al. [38], 2021, Italy	Y	Y	NR	Y	N	NA	NA	NA	Y	NA	Y	NA	NA	N
Cyranka et al., [39], 2021, Poland	Y	Y	NR	Y	N	NA	NA	NA	Y	NA	Y	NA	NA	N
Di Dalmazi et al. [40], Italy	Y	Y	Y	Y	N	NA	NA	NA	Y	NA	Y	NA	NA	Y
Di Riso et al. [41], 2021, Italy	Y	Y	Y	Y	N	NA	NA	NA	Y	NA	Y	NA	NA	Y
Elhenawy and Eltonbary, [42], 2021, Egypt	Y	Y	NR	Y	N	NA	NA	NA	Y	NA	Y	NA	NA	Y
Hosomi et al. [43], 2022, Japan	Y	Y	Y	Y	N	NA	NA	NA	Y	NA	Y	NA	NA	Y
Huang et al. [44], 2022, China	Y	Y	Y	Y	N	NA	NA	NA	Y	NA	Y	NA	NA	N
Kim et al. [45], 2022, US	Y	Y	Y	Y	N	NA	NA	NA	Y	NA	Y	NA	NA	Y
Khari et al. [46], 2021, Iran	Y	Y	NR	Y	Y	NA	NA	NA	Y	NA	Y	NA	NA	N
Madsen et al., [47], 2021, Denmark	Y	Y	Y	Y	N	NA	NA	NA	Y	NA	Y	NA	NA	N
Magliah et al. [48], 2021, Saudi Arabia	Y	Y	NR	Y	N	NA	NA	NA	Y	NA	Y	NA	NA	Y
Munekawa et al. [49], 2021, Japan	Y	Y	Y	Y	N	NA	NA	NA	Y	NA	Y	NA	NA	Y
Miller et al. [50], 2022, US	Y	Y	Y	Y	N	NA	NA	NA	Y	NA	Y	NA	NA	Y
Musche et al., [51], 2021, Germany	Y	Y	NR	Y	N	NA	NA	NA	Y	NA	Y	NA	NA	Y
Myers et al., [52], 2021, US	Y	Y	NR	Y	N	NA	NA	NA	Y	NA	Y	NA	NA	N
Olickal et al. [53], 2020, India	Y	Y	Y	Y	Y	NA	NA	NA	Y	NA	Y	NA	NA	Y
Naous et al. [54], 2022, Lebanon	Y	Y	NR	Y	N	NA	NA	NA	Y	NA	Y	NA	NA	N
Nassar and Salameh, [55], 2021, Lebanon	Y	Y	N	Y	N	NA	NA	NA	Y	NA	Y	NA	NA	N
Regeer et al. [56], 2021, Netherlands	Y	Y	Y	Y	N	NA	NA	NA	Y	NA	Y	NA	NA	Y
Ruissen et al. [57], 2021, Netherlands	Y	Y	NR	Y	N	NA	NA	NA	Y	NA	Y	NA	NA	Y
Sacre et al. [58], 2021, Australia	Y	Y	Y	Y	N	NA	NA	NA	Y	NA	Y	NA	NA	Y
Shin et al. [59], 2021, Korea	Y	Y	Y	Y	N	NA	NA	NA	Y	NA	Y	NA	NA	Y
Silveira et al. [60], 2021, Brazil	Y	Y	NR	Y	N	NA	NA	NA	Y	NA	Y	NA	NA	Y
Sisman et al. [61], 2021, Turkey	Y	Y	NR	Y	N	NA	NA	NA	Y	NA	Y	NA	NA	Y
Utli and Vural Doğru [62], 2021, Turkey	Y	Y	Y	Y	Y	NA	NA	NA	Y	NA	Y	NA	NA	Y

N = No, Q = Question, Y = Yes.
